# Pancreatic Tumors and Immature Immunosuppressive Myeloid Cells in Blood and Spleen: Role of Inhibitory Co-Stimulatory Molecules PDL1 and CTLA4. An *In Vivo* and *In Vitro* Study

**DOI:** 10.1371/journal.pone.0054824

**Published:** 2013-01-24

**Authors:** Daniela Basso, Paola Fogar, Massimo Falconi, Elisa Fadi, Cosimo Sperti, Chiara Frasson, Eliana Greco, Domenico Tamburrino, Sara Teolato, Stefania Moz, Dania Bozzato, Michela Pelloso, Andrea Padoan, Giuseppe De Franchis, Elisa Gnatta, Monica Facco, Carlo-Federico Zambon, Filippo Navaglia, Claudio Pasquali, Giuseppe Basso, Gianpietro Semenzato, Sergio Pedrazzoli, Paolo Pederzoli, Mario Plebani

**Affiliations:** 1 Department of Medicine, University of Padova, Padova, Italy; 2 Chirurgia B, Department of Surgery, University of Verona, Verona, Italy; 3 Department of Surgical, Oncological and Gastroenterological Sciences, University of Padova, Padova, Italy; 4 Department of Women’s and Children’s Health, Laboratory of Pediatric Hemato-Oncology, University of Padova, Padova, Italy; Northwestern University, United States of America

## Abstract

**Background:**

Blood and spleen expansion of immature myeloid cells (IMCs) might compromise the immune response to cancer. We studied *in vivo* circulating and splenic T lymphocyte and IMC subsets in patients with benign and malignant pancreatic diseases. We ascertained *in vitro* whether pancreatic adenocarcinoma (PDAC)-associated IMC subsets are induced by tumor-derived soluble factors and whether they are immunosuppressive focusing on the inhibitory co-stimulatory molecules PDL1 and CTLA4.

**Methodology and Principal Findings:**

103 pancreatic and/or splenic surgical patients were enrolled including 52 PDAC, 10 borderline and 10 neuroendocrine tumors (NETs). Lymphocytes and IMCs were analysed by flow cytometry in blood, in spleen and in three PDAC cell conditioned (CM) or non conditioned PBMC. PDL1 and CTLA4 were studied in 30 splenic samples, in control and conditioned PBMC. IMCs were FACS sorted and co-coltured with allogenic T lymphocytes. In PDAC a reduction was found in circulating CD8^+^ lymphocytes (p = 0.004) and dendritic cells (p = 0.01), which were reduced *in vitro* by one PDAC CM (Capan1; p = 0.03). Blood myeloid derived suppressive cells (MDSCs) CD33^+^CD14^−^HLA-DR^−^ were increased in PDAC (p = 0.022) and were induced *in vitro* by BxPC3 CM. Splenic dendritic cells had a higher PDL1 expression (p = 0.007), while CD33^+^CD14^+^HLA-DR^−^ IMCs had a lower CTLA4 expression (p = 0.029) in PDAC patients. *In vitro* S100A8/A9 complex, one of the possible inflammatory mediators of immune suppression in PDAC, induced PDL1 (p = 0.018) and reduced CTLA4 expression (p = 0.028) among IMCs. IMCs not expressing CTLA4 were demonstrated to be immune suppressive.

**Conclusion:**

In PDAC circulating dendritic and cytotoxic T cells are reduced, while MDSCs are increased and this might favour tumoral growth and progression. The reduced CTLA4 expression found among splenic IMCs of PDAC patients was demonstrated to characterize an immune suppressive phenotype and to be consequent to the direct exposure of myeloid cells to pancreatic cancer derived products, S100A8/A9 complex in particular.

## Introduction

Metastases to distant organs, invasion of adjacent organs and angioinvasion characterize exocrine and endocrine pancreatic tumors [1], [2], the metastatic switch depending on the accumulation of genetic and epigenetic alterations within the tumor cells, which detach from the primary site and migrate into the circulatory system to become embedded in a secondary site [3]. The metastatic cascade, however, does not only reflect the presence of primary tumor cells with a tendency to metastasize; this intricate and complex phenomenon depends on interactions between tumor cells and the adjacent stromal and inflammatory cells, which establish a favourable environment for tumor growth and concur in piloting the migration of tumor cells to distant organs through the release of cytoactive molecules [2]–[5].

Inadequate immune response to cancer cells, a widely debated issue phenomenon, may enable primary tumor growth and metastasis [3]. This failure may depend on the ability of tumors, including pancreatic ductal adenocarcinoma (PDAC), the fourth leading cause of cancer-related death in the US [6], to escape immune recognition and destruction through the loss or down-regulation of the antigen presenting MHC molecules [7], or through the reduction in the capacity of the MHC to complex with antigenic peptides [8]. An ineffective anti-tumor immune response may also depend on dysregulation and functional impairment of immune cells, including CD8^+^, T regulatory lymphocytes (Treg), dendritic cells and myeloid derived cells [9]–[16].

Immature myeloid cells may significantly contribute to tumor progression by inhibiting the adaptive immune response against tumor cells in lymphoid organs, and by migrating to tumor sites where they differentiate into highly immune suppressive tumor associated macrophages [17], [18]. The acronym MDSCs (myeloid derived suppressive cells), a definition based on function, encompasses a myeloid derived heterogenous population of immature myelo-monocytic cells [18], which share the ability to suppress T cells, produce arginase and express inducible nitric oxide synthase (iNOS) [19]. The levels of these cells, phenotypically characterized in the mouse by CD11b and Gr1 markers [18]–[20], are increased in the pancreas, lymph nodes, liver and spleen of pancreatic cancer bearing mice [21]–[26], but only in the spleen (not the pancreas) of mice bearing the pancreatic cancer precursor lesion PanIN [21]. In murine pancreatic cancer models, MDSCs also appear to be relevant factors in causing vaccination and therapy to be ineffective [22], [27], [28], and the spleen appears to be the main organ site for the accumulation of MDSCs [23], [26], [29]. Few data are available in the literature on circulating immature myeloid cells in human PDAC [23], [30]–[32], and the findings made have been compared with those obtained in healthy subjects [31], [32] or patients with gastro-intestinal tract tumors not involving the pancreas [31]. No data are available on the behavior of these cells in humans with endocrine pancreatic tumors and benign pancreatic diseases; nor has the pattern of immature myeloid cells been studied in the human spleen. Although murine MDSCs reliably express the surface markers Gr-1 and CD11b, there is no direct analogs cell surface marker for Gr-1 in humans and numerous subpopulations of immature CD33^+^ and/or CD11b^+^ circulating myeloid cells have been described in different tumors [19], [20], [23], [31]–[35].

Besides arginase and iNOS, tumor-induced MDSCs might overexpress HIF-1α, STAT3, C/EBPβ [23], [29], BCL-2 and VEGFR1 signalling molecules [25], [36] but little is known about the involvement of the inhibitory co-stimulatory molecules PDL1 and CTLA4, important factors contributing to tumor-mediated immune suppression [37]–[43], which blockade by monoclonal antibodies represents an emerging anti-cancer strategy [16], [44], [45]. Among the complex network of cytokines and inflammatory molecules at the tumor stroma interface that fosters MDSCs [8], [29], [31], [46], the S100A8 and S100A9 proteins, expressed by both stromal and tumor cells, appear relevant in the PDAC setting [5], [47], [48].

The main aim of the present study was to contribute to the knowledge on immune suppression in human pancreatic benign and malignant diseases by studying the pattern of circulating and splenic lymphocyte subsets and immature myeloid cells in patients with PDAC, PDAC precursor lesions or pancreatic neuroendocrine tumors (NETs), using subjects with benign cystic adenoma and chronic pancreatitis as controls. Further endpoints were to assess whether pancreatic cancer cells are able to expand MDSCs *in vitro* and to evaluate the role of the inhibitory co-stimulatory molecules PDL1 and CTLA4 searching also for any potential effect of the S100A8 and S100A9 molecules.

## Patients and Methods

### Patients

One-hundred-three consecutive patients (51 males, 52 females, median age: 62 years; age range: 21–83 years) were enrolled in two surgical units for pancreatic diseases (Department of Surgical, Oncological and Gastroenterological Sciences, DiSCOG, University of Padova and Department of Surgery, Chirurgia B, University of Verona, Italy) from November 2009 to April 2012. The study series included patients who underwent i) pancreatoduodenectomy or distal spleno-pancreatectomy for benign or malignant pancreatic disease; ii) splenectomy for non-neoplastic disease. Patients’ diagnoses and respective surgery are listed in Table 1.

**Table 1:** Baseline patients’ characteristics.

Diagnoses	Cases	Age	Surgery			Blood		Spleen	
	(M:F)	(range)	PD	DP	PR	T cells	M cells	T cells	M cells
**PDAC**	52 (31∶21)	70 (48–83)	19	21	12	51	34	20	16
**NETs**	10 (5∶5)	53 (34–76)	0	10	0	10	5	10	8
**BPNs**	10 (2∶8)	49 (21–73)	2	7	1	10	7	8	8
**SCA**	9 (1∶8)	57 (30–83)	1	7*	1	9	0	5	0
**ChrPa**	7 (5∶2)	52 (25–81)	2	2	3	7	4	3	2
**Other tumors** §	9 (4∶5)	67 (54–75)	6	3	0	9	0	0	0
**Benign splenic lesions**	6 (3∶3)	56 (36–76)	6 splenectomies			6	5	5	5

The total number of cases (Cases), the male:female (M:F) ratio, the mean age (years) with minimum and maximum values (range) of patients subdivided according to the histologically confirmed diagnoses, are reported in the first three columns. Surgery indicates the number of cases subjected to pancreatoduodenectomy (PD), distal pancreatectomy (DP) palliative resection (PR). Blood and spleen columns report the number of cases for whom T cells or immature myeloid cells (M cells) subsets were available. PDAC = pancreatic ductal adenocarcinoma; NETs = pancreatic neuroendocrine tumors; BPNs = pancreatic borderline neoplasms; SCA = serous cystadenoma; ChrPa = chronic pancreatitis;

§ Other tumors included 3 papillary, 3 duodenal and 3 stromal tumors.

* 2/7 patients underwent middle pancreatectomies.

Based on the absence or presence of vascular invasion, PDAC patients were divided into two groups: 28 (stage Ia = 1 case, stage Ib = 1, stage IIa = 10 and stage IIb = 16) without and 24 (stage III = 15 cases and stage IV = 9) with vascular invasion.

All patients gave their fully informed consent in writing to participate in the study, which was approved by the local ethics committee (Comitato Etico per la Sperimentazione of the University-Hospital of Padova; Prot. n° 1786P).

### Blood and Spleen Sample Collection and Processing

Before surgery and after overnight fasting, a potassium-EDTA blood sample was collected from each patient. Splenic tissue samples were obtained from patients who underwent distal splenopancreatectomy or splenectomy. After surgical removal, splenic samples (2.5×2.5×1 cm) were crushed and passed through a 100 µm cell strainer (BD Bioscience, San Josè, CA, USA). To obtain single cell suspensions, all samples were repeatedly passed through an 18G syringe and filtered through a 30 µm cell strainer (Partec, Munster, Germany), and then suspended in RPMI 1640 - FCS 10% (Invitrogen, Carlsbad, CA, USA ) for flow cytometry analysis.

### Flow Cytometry Analysis

Flow cytometry data from peripheral blood and splenic cell suspension, acquired using multicolor argon (488 nm) and helium-neon (633 nm) laser cytomics (FC 500 flow cytometer), were analyzed with CXP 2.2 software (Beckman Coulter, Miami, FL, USA). The following monoclonal antibodies were used: CD3-PC5, CD3-ECD, CD4-PE, CD8-ECD, CD45-FITC, CD45-ECD, HLA-DR-PC5, CD33-FITC and CD14-PC7 (Beckman Coulter, Miami, FL, USA); CD25-PE, CD4-FITC, CTLA4-PE (BD Biosciences); CD45-PC5 (Invitrogen); PDL1-PE (eBioscience, San Diego, CA, USA). The antibody panels were: CD4, CD8, CD3, CD45 for CD4^+^ or CD8^+^ T cells; CD4, CD3, CD25, CD45 for CD4^+^CD25^+^ lymphocytes; CD33, HLA-DR, CD45, CD14 for immature myeloid cells; CD33, HLA-DR, CD45, CD14, PDL-1 or CTLA4 for negative co-stimulatory molecules.

### Real Time PCR (RT-PCR) for S100A8 and S100A9 mRNA Quantification

S100A8 and S100A9 mRNA was submitted for relative quantification by means of comparative C_T_ method. Three micrograms of total RNA (High Pure RNA isolation kit, Roche, Monza, Italy) obtained from 9 PDAC splenic samples and from peripheral blood mononuclear cells (PBMC) of healthy blood donors, were used. S100A8 was amplified as described previously [5]. S100A9 was amplified starting with 150 ng cDNA and using the primer pair, 5′GCTCCTCGGCTTTGACAGAGT3′ (S100A9-F) and 5′GCGTTCCAGCTGCGACAT3′ (S100A9-R), and the TaqMan probe, 5′6-FAM-CAAGACGATGACTTGCAA-MGB3′ (S100A9-P) (Applied Biosystems, Monza, Italy). Each sample was amplified in triplicate.

### *In vitro* Experiments

The human BxPC3 (kindly donated by Dr. Andrea Galli, University of Florence, Italy), Capan1 and MiaPaCa2 (American Type Culture Collection) pancreatic cancer cell lines were used. 4×10^5^ Capan1 and 2×10^5^ BxPC3 and MiaPaCa2 cells were seeded in 75-cm^2^ flasks with 15 mL RPMI (Invitrogen) with added 0.1% gentamycin (Invitrogen) and 1% FCS (Invitrogen) and kept in continuous culture at 37°C in a humidified atmosphere (5% CO_2_) for four days. The culture media (conditioned media) were then collected, adjusted to 10% FCS, and used for the experiments with peripheral blood mononuclear cells (PBMC) within two hours after collection.

Human PBMC were isolated from a total of 40 blood donors’ buffy coats by differential density gradient centrifugation (Histopaque®-1077, Sigma-Aldrich, Milano, Italy). In the first series of experiments, PBMC from 17 donors were split into two or more fractions and cultured for four days (6×10^6^ cells in each well of a 6 well culture plate) in complete control medium (RPMI, 10% FCS), or in pancreatic cancer cell conditioned media. After collection, the cells were analyzed under flow cytometry. PBMC from 7 donors were split into two fractions and cultured for two days (6×10^6^ cells in each well of a 6 well culture plate) in complete control medium (RPMI, 10% FCS) in the absence or presence of 10 nM S100A8/A9 complex (DBA Italia srl, Milano, Italy) and then analysed by flow cytometry. In the second series of experiments, PBMC from 7 blood donors were cultured for 4 days in control and Capan1 conditioned media. After collection 50×10^6^ cells were incubated in the dark for 30 minutes with 15 µL CD33-FITC, 20 µL HLA-DR-PC5, 15 µL CD14-PC7, 15 µL CD45-ECD. Cells that were CD33^+^CD14^−^HLA-DR^+^ were sorted (BD FACSAria III**,** BD Biosciences, San Jose, CA, USA), seeded in a 96 well plate and co-cultured with 50,000 allogenic total T lymphocytes (RosetteSep kit, StemCell Technologies, Voden Medical Instruments Spa, Milano, Italy), in 1∶20 and in 1∶40 ratio. Each experiment was run with three different allogenic total T lymphocytes. Total T lymphocytes proliferation was determined on the basis of (^3^H)-thymidine uptake. In the third series of experiments Capan1 conditioned PBMC from 4 donors were used. CD33^+^CD14^−^HLA-DR^+^PDL1^+^ and CD33^+^CD14^−^HLA-DR^+^PDL1^−^ were sorted, co-cultured with three different allogenic total T lymphocytes in a 1∶20 ratio in the above-described conditions. For each donor/allogenic total T lymphocyte combination, we calculated the mean of three measurements obtained from total T proliferation of cells co-cultured with CD33^+^CD14^−^HLA-DR^+^PDL1^−^ and with CD33^+^CD14^−^HLA-DR^+^PDL1^+^. A series of 11 results were paired to perform the statistical analysis. In the fourth series of experiments, dendritic cells (DCs) from human peripheral blood CD14^+^ monocytes from 5 blood donors’ buffy coats were obtained by negative selection (RosetteSep kit, StemCell Technologies, Voden Medical Instruments Spa, Milano, Italy) and gradient centrifugation. 2.5×10^6^ CD14^+^ cells were plated in each well of a 6-well plate and cultured for seven days in RPMI in 10% FCS supplemented with 50 ng/mL GM-CSF (PeproTech, DBA Italia srl, Segrate, Italy) and 50 ng/mL IL4 (PeproTech) to obtain DCs. The medium was changed every second day by removing one third of the medium and adding freshly made medium supplemented with cytokines. To obtain mature DCs, 100 ng/mL LPS (Sigma-Aldrich) were added on day seven, and cells were cultured for 48 hours before collection. CD14^−^HLA-DR^+^CTLA4^+^ and CD14^−^HLA-DR^+^CTLA4^−^ were sorted, co-cultured with three different allogenic total T lymphocytes in a 1∶20 ratio in the above-described conditions.

### Statistical Analysis

The statistical analysis of data was made using the non parametric Kruskal-Wallis test, Wilcoxon signed ranks test, multiple comparison between groups with adjusted p-value, Cox proportional hazard model, non parametric test for trend across ordered groups, the Chi-Square test, and Spearman’s correlation, SPSS 9.0 and Stata (Statacorp, Texas, USA) statistical softwares being employed. Patients with splenic non-neoplastic lesions (6 cases) and those with chronic pancreatitis (7 cases) were considered overall as the reference group.

## Results

Patients’ details are reported in Table 1. The demographic characteristics, the histologically confirmed diagnoses and the surgical procedures are shown together with the number of cases in which T cells and immature myeloid cells subsets were analyzed and were available in blood and spleen samples.

### CD8^+^ T cells are Reduced in the Peripheral Blood of Patients with Pancreato-biliary Tract Tumors

**Figure 1:**
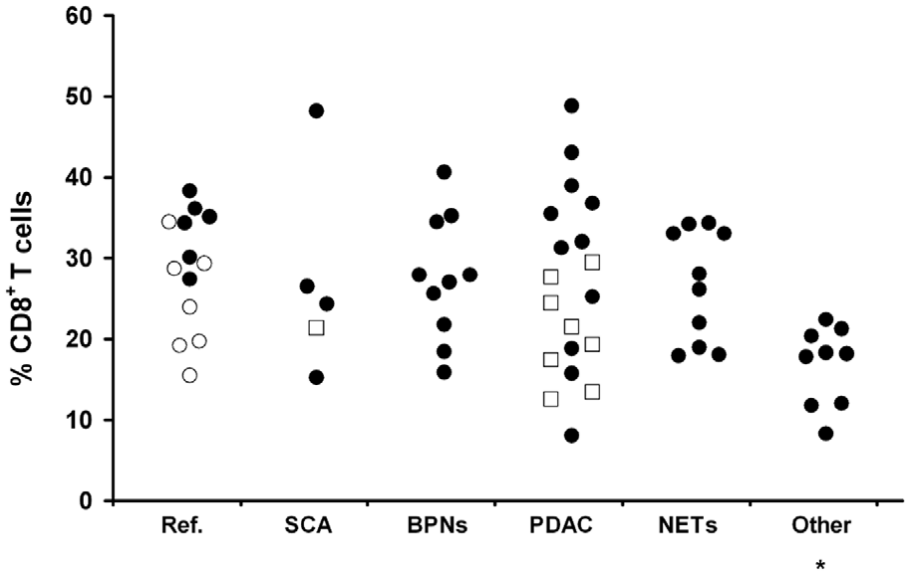
Individual levels of CD8^+^ T cells in blood of the studied patients. Ref. = reference group made of patients with chronic pancreatitis (open dots) and of patients with splenic non-neoplastic lesions; SCA = Serous cystadenoma; BPNs = Borderline pancreatic neoplasms; PDAC = Ductal adenocarcinoma; NETs = Neuroendocrine tumors; Other = Non-pancreatic tumors. Each dot represents one case, and each open square represents five cases. * = p<0.0001 with respect to Ref. and p<0.001 with respect to BPNs.

No differences were found between patients for circulating and splenic CD4^+^ (Kruskal-Wallis test: p = 0.906 and p = 0.378) and CD4^+^CD25^+^ T cells (p = 0.596 and p = 0.420) or splenic CD8^+^ T cells (p = 0.290). On the contrary circulating CD8^+^ T cells significantly varied between groups (Kruskal-Wallis test: p = 0.004)(Figure 1). Although PDAC patients had lower CD8^+^ T cells than reference patients, this difference was not powerful enough to reach the statistical significance (p = 0.006, adjusted p value for significance = 0.002), while significantly lower levels were found in patients with other tumors with respect to reference patients (p = 0.0001) and borderline pancreatic neoplasms (p = 0.001).

### CD14^−^HLA-DR^+^ Dendritic Cells are Reduced and CD14^−^HLA-DR^−^ MDSCs are Increased in the Peripheral Blood of PDAC Patients

**Figure 2:**
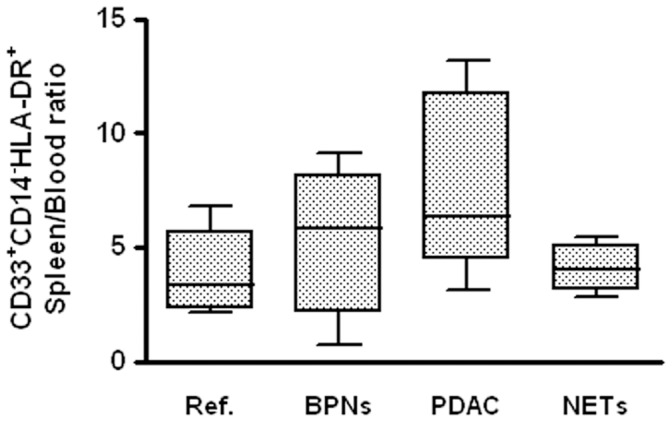
Ratio between splenic and circulating CD33^+^CD14^−^HLA-DR^+^ immature myeloid cells. Ref. = reference group made of patients with chronic pancreatitis and of patients with splenic non-neoplastic lesions; BPNs = Borderline pancreatic neoplasms; PDAC = Ductal adenocarcinoma; NETs = Neuroendocrine tumors. Boxes represent interquartile ranges with medians; bars represents minimum and maximum values.

Immature circulating and splenic myeloid cells were gated on the basis of CD33 expression levels, excluding lymphocytes and mature granulocytes. Depending on CD14 and HLA-DR expression levels, CD33^+^ cells were classified in four subsets: CD14^+^HLA-DR^+^, CD14^+^HLA-DR^−^, CD14^−^HLA-DR^+^ and CD14^−^HLA-DR^−^. Only CD14^−^HLA-DR^+^ circulating dendritic cells were correlated with disease diagnosis (Kruskal-Wallis Test: p = 0.01), lower levels being found in PDAC than in NETs (p = 0.003, adjusted p value for significance = 0.004). In spleen these cells tended to be higher in PDAC, NETs and borderline pancreatic tumors than in the reference cases (p = 0.077). The ratio between splenic and circulating CD14^−^HLA-DR^+^ was slightly higher in borderline tumors, but significantly higher in PDAC cases than in reference (Test for trend across ordered groups: p = 0.028) (Figure 2).

**Figure 3:**
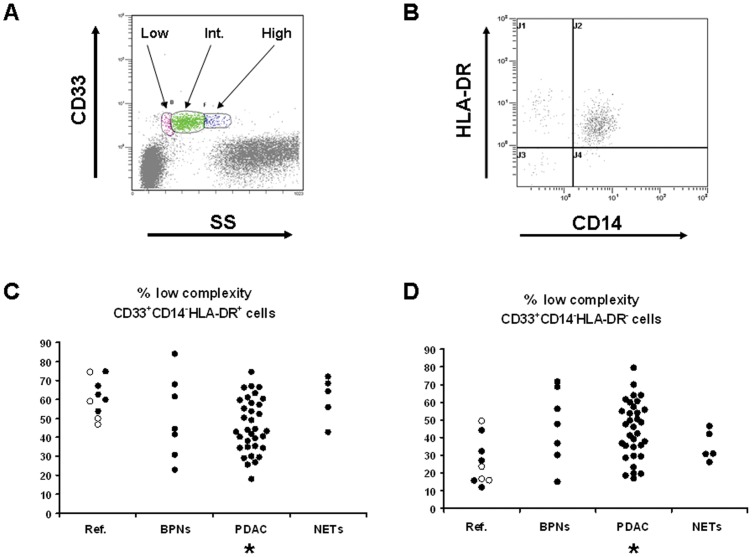
Immature myeloid cells in peripheral blood. Panel A (upper left): a typical example of gating of low, intermediate (Int.) and high complexity sets among CD33^+^ cells in flow cytometry. Panel B (upper right): low, intermediate and high complexity CD33^+^ cells were analysed on the basis of CD14 and HLA-DR expression. A typical example is shown in this panel. Panel C (lower left): Blood low complexity CD33^+^CD14^−^HLA-DR^+^ cells. Panel D (lower right): blood low complexity CD33^+^CD14^−^HLA-DR^−^ cells. Ref. = reference group made of patients with chronic pancreatitis (open dots) and of patients with splenic non-neoplastic lesions; BPNs = Borderline pancreatic neoplasms; PDAC = Ductal adenocarcinoma; NETs = Neuroendocrine tumors. * = p<0.004 (adjusted p-value for significance) with respect to Reference.

The cellular complexity that CD33^+^ cells presented at further evaluation was taken into account for a complete analysis of results. Three sets with low, intermediate and high complexity found in the whole CD33^+^ blood and splenic cell population are shown in Figure 3 (panel A). As above, in each of the three sets, four subsets were identified (Figure 3, panel B).

None of the four circulating immature myeloid cells subsets with intermediate or high complexity correlated with disease diagnosis (p>0.05 for all eight subsets). Among low complexity circulating immature myeloid cells, CD14^−^HLA-DR^+^ were significantly reduced (Kruskal-Wallis test: p = 0.026; Figure 3, panel C), whereas low complexity CD14^−^HLA-DR^−^ MDSCs were significantly increased in PDAC with respect to the reference group (Kruskal-Wallis test: p = 0.022; Figure 3, panel D). In the spleen, none of the low, intermediate or high complex subset was correlated with the disease diagnosis.

### Increased CD14^+^HLA-DR^−^ MDSCs in the Peripheral Blood and Spleen of PDAC Patients Correlate with Vascular Invasion

PDAC patients, subdivided based on the presence (n = 24) or absence (n = 28) of vascular invasion, had a median post-operative follow-up of 12.5 (range 1–23) months during which 18 patients died of disease-related causes.

Vascular invasion was significantly associated with a worse prognosis, as reported in Table 2.

**Table 2:** Cox regression analysis of vascular invasion corrected for age and gender for survival in PDAC patients.

	HR	95% CI	p =
**Age**	1.01	0.94–1.09	0.649
**Gender**	0.96	0.31–3.03	0.953
**Vascular invasion**	6.04	1.62–22.54	0.007

HR = Hazard ratio; CI = confidence interval.

The studied circulating and splenic CD4^+^, CD8^+^ and CD4^+^CD25^+^ lymphocyte subsets were not correlated with the presence or absence of vascular invasion (p>0.05 for all subsets) and nor were they predictive of overall survival after surgery (Cox proportional hazard model corrected for age and sex: p>0.05 for all subsets).

Table 3 reports median values, interquartile ranges and a statistical analysis (Kruskal-Wallis test) for circulating and splenic immature myieloid cell subsets in PDAC patients with or without vascular invasion at diagnosis. Circulating CD14^+^HLA-DR^+^ cells were reduced, while both circulating and splenic CD14^+^HLA-DR^−^ MDSCs were increased in PDAC patients with vascular invasion.

**Table 3:** Circulating (blood) and splenic immature myeloid cell subsets in PDAC patients subdivided according to the presence (Yes) or absence (No) of vascular invasion.

		No (n = 16)	Yes (n = 11)	
		Median (IQR)	Median (IQR)	p-value
**Blood**	**CD14^+^HLA-DR^+^**	84.2 (76.5–88.1)	74.8 (71.4–83.4)	0.008
	**CD14^+^HLA-DR** ^−^	1.5 (0.4–3.9)	7.7 (2.1–21.9)	0.022
	**CD14** ^−^ **HLA-DR^+^**	8.5 (6.2–12.5)	10.4 (6.1–13.7)	0.961
	**CD14** ^−^ **HLA-DR** ^−^	4.6 (3.2–8.3)	4.3 (3.3–6.2)	0.693
		**No (n = 7)**	**Yes (n = 5)**	
		**Median (IQR)**	**Median (IQR)**	**p-value**
**Spleen**	**CD14^+^ HLA-DR^+^**	27.7 (18.2–48.7)	35.9 (31.2–52.9)	0.223
	**CD14^+^HLA-DR** ^−^	0.0 (0.0–0.4)	0.6 (0.2–2.2)	0.028
	**CD14** ^−^ **HLA-DR^+^**	66.8 (47.4–69.9)	57.2 (41.4–58.8)	0.223
	**CD14** ^−^ **HLA-DR** ^−^	5.9 (2.2–10.3)	6.2 (3.3–10.3)	0.935

Median values, interquartile range (IQR) and a statistical analysis (Kruskal-Wallis test) are reported.

### Splenic Immature Myeloid Cells Express High PDL1 and Low CTLA4 in PDAC Patients. PDL1 Expression Correlates with S100A8 and S100A9 mRNA

**Figure 4:**
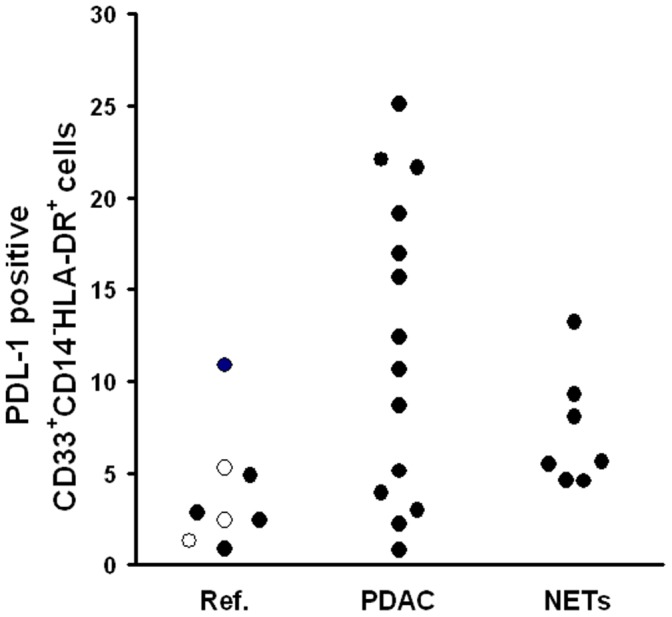
Percentage of PDL-1 expression among splenic CD33^+^CD14^−^HLA-DR^+^ cells. Ref. = reference group made of patients with serous cystadenoma (open dots) and of patients with splenic non-neoplastic lesions; PaCa = Ductal adenocarcinoma; NETs = Neuroendocrine tumors. Kruskal-Wallis test: p = 0.046.

In a series of 30 splenic samples (5 reference, 3 borderline tumors, 15 PDAC, 7 NETs), we studied the membranal expression of the two inhibitory co-stimulatory molecules PDL1 and CTLA4. PDL1 was highly expressed by immature CD14^−^HLA-DR^+^ cells in PDAC with respect to reference patients (p = 0.007, adjusted p value for significance = 0.008)(Figure 4), while low CTLA4 expression (less than 15% positive cells) was found among CD14^+^HLA-DR^−^ cells in the majority of PDAC patients (13/15, 86.6%) differently from all the other studied patients (5/16, 31.3%), and this difference was statistically significant (chi-square = 9.05; p = 0.029).

The fold increase of S100A8 and S100A9 mRNA, analysed in a series of 9 PDAC splenic samples, varied from 0 to 22 for S100A8 (median: 2.2, interquartile range: 0.6–5.7) and from 0 to 11.3 for S100A9 (median: 2.1, interquartile range: 0.5–3.4). S100A8 and S100A9 mRNA expression levels were correlated each other (Spearman’s r = 0.946, p<0.0001). Both S100A8 and S100A9 were correlated with the percentage of CD14^+^HLA-DR^+^PDL^+^ immature myeloid splenic cells (r = 0.819, p<0.001 for both), but not with the other studied lymphocyte and immature myeloid cell subsets.

### Pancreatic Cancer Cell Lines Conditioned Media Restrict CD14^−^HLA-DR^+^ Dendritic Cell and Expand CD14^−^HLA-DR^−^ MDSCs

PBMC from 17 healthy donors were analysed by flow cytometry to identify lymphocyte and immature myeloid cell subsets after they have been cultured for 4 days in control, BxPC3, Capan1 or MiaPaCa2 conditioned media. Table 4 reports median, interquartile ranges and a statistical analysis of data. The percentage of CD4^+^CD25^+^ lymphocytes was higher, while the percentage of CD14^−^HLA-DR^+^ dendritic cells was lower in Capan1 conditioned than in control PBMC; BxPC3 conditioned media induced the expansion of CD14^−^HLA-DR^−^ MDSCs.

**Table 4:** Pancreatic cancer cell conditioned media effects on lymphocyte and immature myeloid cell subsets.

	Control (n = 17)	BxPC3 (n = 6)	Capan1 (n = 11)	MiaPaCa2 (n = 6)
	Median (IQR)	Median (IQR)	Median (IQR)	Median (IQR)
**CD4^+^**	50 (44–57)	58 (39–61)	50 (41–51)	56 (41–61)
**p-value**		0.674	0.386	0.917
**CD8^+^**	23 (15–27)	19 (15–31)	22 (16–27)	19 (15–32)
**p-value**		0.715	0.625	0.917
**CD4^+^CD25^+^**	10 (9–11)	9 (8–11)	12 (10–13)	9 (8–11)
**p-value**		0.269	0.014*	0.599
**CD14^+^HLA-DR^+^**	71 (67–86)	59 (36–74)	81 (76–88)	59 (40–77)
**p-value**		0.075	0.062	0.463
**CD14^+^HLA-DR** ^−^	0.0 (0.0–0.4)	0.2 (0.0–0.3)	0.1 (0.0–0.4)	0.0 (0.0–0.3)
**p-value**		0.465	0.391	0.715
**CD14** ^−^ **HLA-DR^+^**	16.5 (8–21)	26.9 (15–47)	10.8 (10–14)	29.7 (12–46)
**p-value**		0.116	0.033*	0.249
**CD14** ^−^ **HLA-DR** ^−^	9.3 (3–13)	14.3 (12–16)	6.7 (1–11)	9.7 (8–17)
**p-value**		0.028*	0.285	1.00

A total of 17 healthy PBMC were analysed by flow cytometry after they have been cultured for 4 days in control medium or pancreatic cancer cell conditioned media. PBMC from 11 donors were cultured in Capan1 conditioned and in their respective control media, while PBMC from 6 donors were cultured in BxPC3 and MiaPaCa2 conditioned media and in their respective control media. The median and interquartile ranges (IQR) of the percentage of lymphocyte and CD33^+^ immature myeloid cell subsets are shown. The statistical analysis of data (Wilcoxon signed rank test) was made by pairing any conditioned media result with its own control. Asterisks highlight statistical significance.

CD14^−^HLA-DR^+^ dendritic cells from control and Capan1 conditioned PBMC obtained from a separate series of 8 healthy donors, were FACS sorted and co-cultured with allogenic T cells to assess their suppressive function. The proliferation of allogenic T cells co-cultured with control or Capan1 conditioned CD14^−^HLA-DR^+^ cells did not significantly differ when CD14^−^HLA-DR^+^/T cell ratio was at 1∶40 (Wilcoxon singed ranks test: p = 0.81) nor when it was 1∶20 (p = 0.54).

### Pancreatic Cancer Cell Lines Conditioned Media and the S100A8/S100A9 Complex Induce PDL1 and Reduce CTLA4 Expression in Immature Myeloid Cells

**Figure 5:**
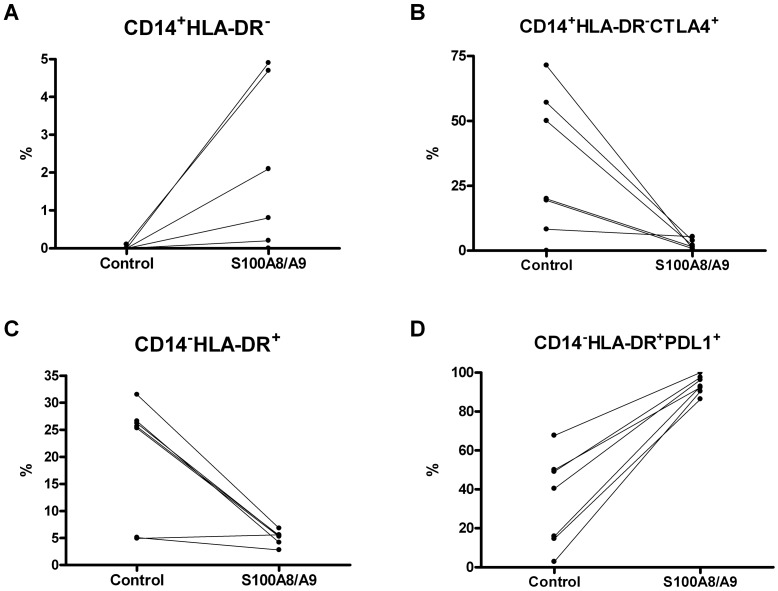
S100A8/A9 induces PDL1 and inhibits CTLA4. Healthy PBMC were analysed by flow cytometry after they have been cultured for 2 days in the absence (Control) or presence of 10 nM S100A8/A9 heterocomplex. Immature myeloid cells were gated on the basis of CD33 expression. Panel A: percentage variations of CD14^+^HLA-DR^−^ MDSCs; panel B: percentage variations of CTLA4 among CD14^+^HLA-DR^−^ MDSCs; panel C: percentage variations of CD14^−^HLA-DR^+^ dendritic cells; panel D: percentage variations of PDL1 among CD14^−^HLA-DR^+^ dendritic cells.

We then evaluated the percentage of PDL1 and CTLA4 positive cells among myeloid cell subsets in control and pancreatic cancer cell conditioned PBMC and results are shown in Table 5. Capan1 conditioned media caused a significant increase of PDL1 among CD14^+^HLA-DR^+^ and CD14^−^HLA-DR^+^ immature myeloid cells, and a significant reduction in the percentage of CTLA4 positive cells among all myeloid cell populations. BxPC3 conditioned media caused a similar reduction in CTLA4 positive cells among those CD14^−^HLA-DR^−^. The treatment of PBMC with the S100A8/A9 heterocomplex caused a significant increase in the percentage of CD14^+^HLA-DR^+^ (Wilcoxon signed ranks test: p = 0.028) and of CD14^+^HLA-DR^−^ (p = 0.043) (Figure 5, panel A) paralleled by a significant reduction in the percentage of CD14^−^HLA-DR^+^ (p = 0.017) myeloid cells (Figure 5, panel C). S100A8/A9 treatment caused also a significant enhancement of PDL1 positive cells among CD14^+^HLA-DR^+^ (p = 0.018), CD14^−^HLA-DR^+^ (p = 0.018) (Figure 5, panel D) and CD14^−^HLA-DR^−^ (p = 0.043) subsets and a significant reduction of CTLA4 positive cells among those CD14^+^HLA-DR^−^ (p = 0.028) (Figure 5, panel B).

**Table 5:** Pancreatic cancer cell conditioned media effects on PDL1 and CTLA4 in immature myeloid cell subsets.

	Control (n = 14)	BxPC3 (n = 6)	Capan1 (n = 8)	MiaPaCa2 (n = 6)
**CD14^+^HLA-DR^+^PDL^+^**	56 (37–73)	61 (23–82)	76 (73–92)	50 (21–76)
**p-value**		0.249	0.036*	0.686
**CD14** ^−^ **HLA-DR^+^PDL^+^**	31 (22–36)	26 (10–50)	46 (39–53)	33 (28–45)
**p-value**		0.753	0.017*	0.173
**CD14** ^−^ **HLA-DR** ^−^ **PDL^+^**	2.7 (0.4–4.8)	3.3 (0.3–6.3)	3.8 (1.3–10.6)	1.7 (1.3–5.8)
**p-value**		0.753	0.108	0.345
	**Control (n = 12)**	**BxPC3 (n = 6)**	**Capan1 (n = 6)**	**MiaPaCa2 (n = 6)**
**CD14^+^HLA-DR^+^CTLA4^+^**	16 (12–23)	28 (9–54)	6 (1–8)	15 (10–20)
**p-value**		0.345	0.028*	0.500
**CD14** ^−^ **HLA-DR^+^CTLA4^+^**	13 (4–21)	4 (2–16)	4 (1–7)	10 (2–57)
**p-value**		0.116	0.043*	0.917
**CD14** ^−^ **HLA-DR** ^−^ **CTLA4^+^**	12.3 (10.0–13.2)	5.7 (0.5–10.7)	4.3 (2.9–5.8)	8.3 (2.9–19.5)
**p-value**		0.028*	0.046*	0.753

Healthy PBMC were analysed by flow cytometry after they have been cultured for 4 days in control medium or in pancreatic cancer cell conditioned media. PBMC from 8 donors were cultured in Capan1 conditioned and in their respective control media, while PBMC from 6 donors were cultured in BxPC3 and MiaPaCa2 conditioned media and in their respective control media. In the series of 8 PBMC donors used for Capan1 experiments, CTLA4 data was available for a subset of 6 donors. Median and interquartile ranges (in brackets) of the percentage of CD33^+^ immature myeloid cells expressing PDL1 or CTLA4 are shown. Only few events among the CD33^+^CD14^+^HLA-DR^−^ cell population were obtained (see table 2) and this did not allow an accurate analysis of this subset, which was omitted from the table. The statistical analysis of data (Wilcoxon signed rank test) was made by pairing any conditioned media result with its own control. Asterisks highlight statistical significance.

### CTLA4 Negative Dendritic Cells Inhibit in vitro T-cell Proliferation

**Figure 6:**
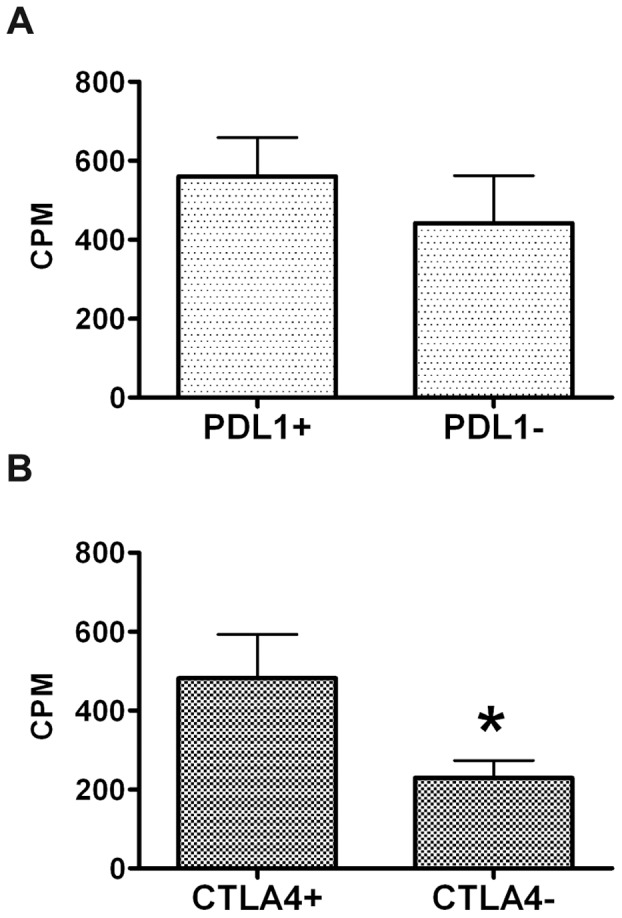
CTLA4 negative dendritic cells suppress T cell proliferation. Panel A: CD33^+^CD14^−^HLA-DR^+^PDL1^+^ and CD33^+^CD14^−^HLA-DR^+^PDL1^−^ cells were FACS sorted and cocultured with allogenic T cells and proliferation was evaluated by (^3^H)-thymidine incorporation. Assay was performed in triplicate; data are mean ± SE of 4 independent experiments. Panel B: CTLA4^+^ and CTLA4^−^ dendritic cells were FACS sorted and cocultured with allogenic T cells and proliferation was evaluated by (^3^H)-thymidine incorporation. Assay was performed in triplicate; data are mean ± SE of 3–4 independent experiments.

FACS sorted CD33^+^CD14^−^HLA-DR^+^PDL1^+^ cells did not significantly modify allogenic T cells proliferation with respect to CD33^+^CD14^−^HLA-DR^+^PDL1^−^ cells (Wilcoxon singed ranks test: p = 0.11), despite they had a slight stimulatory effect (Figure 6, panel A). On the contrary dendritic cells that were CD14^−^HLA-DR^+^CTLA4^−^ inhibited T cell proliferation of about 50% with respect to their CTLA4 positive counterpart (Figure 6, panel B) and this difference was statistically significant (Wilcoxon singed ranks test: p = 0.008).

## Discussion

A dysregulation of myeloid and of T cells is now considered an emerging hallmark of cancer [3]. In the present study an analysis was made of representative subsets of T lymphocyte and of immature myeloid cells in the peripheral blood of patients with pancreatic diseases and, for the first time, in the human spleen. Among blood and spleen lymphocyte subsets, only blood CD8^+^ T cells were significantly lower in PDAC. However, CD8^+^ T cells reduction was not PDAC specific, since it was also found in patients with duodenal, papillary and stromal tumors. We then ascertained whether MDSCs and dendritic cells, which contribute to immune dysfunction, are specifically induced and/or repressed in PDAC patients.

Immature myeloid cells were selected by gating in the first line CD33 positive cells; four different subclasses were then defined on the basis of their high or low expression of the monocytic marker CD14, and of HLA-DR, a marker of mature dendritic cells [49]. The immature CD14^−^HLA-DR^+^ dendritic cells [33], [49], [50] were increased in the spleen of patients with PDAC, NETS and borderline tumors, but they were significantly reduced in blood only in patients with PDAC. Intriguingly, dendritic cells splenic/blood ratio correlated with PDAC progression, being progressively higher in borderline pancreatic tumors and PDAC than in reference. Taken together these findings suggest that only PDAC and the correlated, although less aggressive, borderline tumors, not pancreatic NETs, may impact on the anti-tumor immune response by inhibiting or delaying the migration of dendritic cells from spleen into blood but also by directly reducing their number in the circulation possibly through the release of bioactive molecules.

While the immature myeloid lineage marker CD33 is often taken into consideration for defining MDSCs [30], [51], [52] most other markers for the phenotypic characterization of these cells are heterogeneous and sometimes contrastive [23], [29], [30], [51], [52], [53]. From a morphological viewpoint, immature myeloid cells do not constitute a homogeneous population, but may resemble lymphocytes and macrophages [26], [33]. We therefore focused our study not only on membranal markers, but also on cellular complexity, comprising low, intermediate and high complexity cellular populations. Disease correlated variations were found only among circulating low complexity sets: in PDAC patients CD14^−^HLA-DR^+^ dendritic cells were reduced and associated with a contextual increase in CD14^−^HLA-DR^−^ MDSCs, which are very close to the lin^−/Lo^ HLA-DR^−^CD33^+^CD11b^+^ cells studied by Diaz-Montero et al. in solid tumors and demonstrated to impair T cell activation [52].

Among immature myeloid cells, those CD14^+^HLA-DR^−^ are also considered MDSCs [30], [51], [53] and they were increased in our PDAC patients both in blood and in spleen in the presence of vascular invasion. Since vascular invasion is considered the main predictor for PDAC survival, and this was confirmed also in the present study, we may argue that the expansion of CD14^+^HLA-DR^−^ MDSCs in blood and spleen probably contribute in favoring PDAC progression, thus suggesting that these cells are good candidates for new therapeutic targeting.

Among the incoming cancer immunotherapy strategies, antibody blockade of the inhibitory molecules CTLA4 and PDL1 appears a promising approach [45]. CTLA4 expression and function in T cells is well known and reported to down-modulate the initial stages of T cells activation [54]. This molecule, however, is also expressed by dendritic cells and its engagement is reported to prevent T-cell responses [55]. PDL1, one of the two PD1 ligands, is expressed by both tumor cells and by cells of the tumor microenvironment [56]: its expression by tumor cells promotes neoplastic growth and appears to be of prognostic relevance in pancreatic cancer [38], while its expression on antigen presenting cells in the tumor microenviroment can induce T cell apoptosis [37]. We found higher expression levels of PDL1, not CTLA4, in the splenic dendritic cells of patients with PDAC, in agreement with findings made by Tjomsland et al. [35], who described high levels of PDL1 expression on blood dendritic cells in patients with pancreatic cancer, but not in those with chronic pancreatitis. Moreover the CD14^+^HLA-DR^−^ MDSCs in the spleen of patients with PDAC had a reduced expression of CTLA4. To obtain some mechanicistic insights linking PDAC, myeloid cell subsets, CTLA4 and PDL1, but also to verify whether a different expression of these inhibitory molecules is associated with a different cellular phenotype, *in vitro* experiments were performed using conditioned media from three different pancreatic cancer cell lines. MiaPaCa2 conditioned media did not alter PBMC phenotype nor they affected PDL1 or CTLA4 expression. BxPC3 and Capan1 conditioned media had some different and some similar effects: in Capan1 educated PBMC, CD4^+^CD25^+^ Treg cells increased and CD14^−^HLA-DR^+^ dendritic cells decreased with respect to control PBMC, while in BxPC3 educated PBMC an expansion of CD14^−^HLA-DR^−^ MDSCs was observed. Both BxPC3 and Capan1 conditioned media caused a reduced CTLA4 expression among CD14^−^HLA-DR^−^ MDSCs, while only Capan1 conditioned media reduced CTLA4 expression in the other immature myeloid cell subsets and induced PDL1. Differences and similarities in the effects of pancreatic cancer cell lines on PBMC probably reflect differences and similarities in their genetic make-up, miR and proteomic profiling, and in synthesis and release of cytokines. We have previously demonstrated that Capan1 cells produce much more TG-β1, IL8 and GM-CSF than MiaPaCa2 [57], and that Capan1 and BxPC3, not MiaPaCa2, express high levels of S100A8 [5] and S100A9 [not shown]. These latter molecules, suggested to be involved in favoring MDSCs expansion [47], appeared suitable candidates in our model and their effects were therefore tested *in vitro*.S100A8/A9 complex mimicked the effects of BxPC3 and of Capan1: it caused a reduction of dendritic cells and an expansion of MDSCs, supporting previous findings [47], [58]. S100A8/A9 complex also induced PDL1 and down-regulated CTLA4, and this has never been previously reported. The existence of an association between S100A8/A9 and PDL1 was further supported by the correlation found between S100A8/S100A9 mRNA levels and the percentage of CD14^+^HLA-DR^+^PDL1^+^ cells in human PDAC splenic samples. However, besides S100A8/A9 complex, other PDAC derived molecules might be involved in altering immune cell phenotype. Capan1 differ from BxPC3 and MiaPaCa2 in genetics and membrane biomarkers [59], protein and miR profiling [60], [61]. Of interest is the observation that Capan1, not BxPC3 or MiaPaCa2, release high amounts of GDF15 protein [60] and express high levels of miR 190 [61]: the former protein is potentially involved in tolerance induction [62] and the latter miR in the regulation of MDCSs recruitment [63].

We then evaluated whether variations in PDL1 and CTLA4 expression are associated with an immunesuppressive phenotype. PDL1 expression characterized an overall stimulatory, not inhibitory, phenotype. To study whether CTLA4 is involved in immune suppression, experiments were performed using allogenic T cells co-cultured with dendritic cells. Dendritic cells were chosen for the experiments to obtain representative numbers of cellular events with positive or negative CTLA4 expression but also because dendritic cells are obtained by stimulating PBMC with GM-CSF, a cytokine recently demonstrated to be highly expressed by pancreatic cancer and necessary and sufficient for *in vitro* generation of functional immunosuppressive Gr-1^+^CD11b^+^ cells in mice [26]. Interestingly those dendritic cells lacking the CTLA4 receptor were shown to reduce T cell proliferation and this supports CTLA4 as a candidate molecule to characterize the immunosuppressive phenotype of myeloid cells in humans.

In conclusion, in PDAC clinical setting an overall pattern of immune suppression prevails, characterized by reduced levels of circulating CD8^+^ T cells and dendritic cells and by increased circulating and splenic levels of MDSCs. A reduced expression of CTLA4 among splenic MDSCs was observed in PDAC patients and was demonstrated to be consequent to the direct exposure of myeloid cell to pancreatic cancer derived products and in particular to the S100A8/A9 complex. A reduced CTLA4 expression was also shown to be a feature of an immune suppressive phenotype and this suggests caution in the use of anti-CTLA4 therapies.
